# Categories of Auditory Performance and Speech Intelligibility Ratings of Early-Implanted Children without Speech Training

**DOI:** 10.1371/journal.pone.0053852

**Published:** 2013-01-21

**Authors:** Huiqun Zhou, Zhengnong Chen, Haibo Shi, Yaqin Wu, Shankai Yin

**Affiliations:** 1 Department of Otolaryngology, Affiliated Sixth People's Hospital to Shanghai Jiao Tong University, Shanghai, China; 2 Otolaryngology Institute, Shanghai Jiao Tong University, Shanghai, China; UNLV, United States of America

## Abstract

**Objective:**

To assess whether speech therapy can lead to better results for early cochlear implantation (CI) children.

**Patients:**

A cohort of thirty-four congenitally profoundly deaf children who underwent CI before the age of 18 months at the Sixth Hospital affiliated with Shanghai Jiaotong University from January 2005 to July 2008 were included. Nineteen children received speech therapy in rehabilitation centers (ST), whereas the remaining fifteen cases did not (NST), but were exposed to the real world, as are normal hearing children.

**Methods:**

All children were assessed before surgery and at 6, 12, and 24 months after surgery with the Categories of Auditory Performance test (CAP) and the Speech Intelligibility Rating (SIR). Each assessment was given by the same therapist who was blind to the situation of the child at each observation interval. CAP and SIR scores of the groups were compared at each time point.

**Results:**

Our study showed that the auditory performance and speech intelligibility of trained children were almost the same as to those of untrained children with early implantation. The CAP and SIR scores of both groups increased with increased time of implant use during the follow-up period, and at each time point, the median scores of the two groups were about equal.

**Conclusions:**

These results indicate that great communication benefits are achieved by early implantation (<18 months) without routine speech therapy. The results exemplify the importance of enhanced social environments provided by everyday life experience for human brain development and reassure parents considering cochlear implants where speech training is unavailable.

## Introduction

Cochlear implantation (CI) already has an established role as a treatment for profound hearing impairment. Candidacy for CI has changed gradually but significantly since the first multichannel devices were implanted in the late 1970s. Increasing experience with CI has resulted in children of younger ages being considered as CI candidates [1], [2]. The earliest age of approved cochlear implantation was reduced by the US Food and Drug Administration from 24 months in 1990 to 18 months in 1998 and to 12 months in 2000.

Many factors have been found to affect the outcome of implantation, such as duration of deafness [3], age at onset of deafness and age at implantation [4], [5], duration of implant use, length of daily device use [6], and preoperative level of residual hearing [7]. Although not all reports are in agreement on the effects of these variables [8], all appear to agree that earlier implantation produces better outcomes [9]. Most children implanted before the age of 2 years are able to develop speech and language at a rate equal to similarly aged children with normal hearing [10]. More and more children have received cochlear implants before 2 and even at 1 year of age to obtain better results.

Although the overall outcome for patients with cochlear implants has steadily improved with advances in cochlear implant technology, some patients receive little benefit from the latest cochlear implant technology even after many years of daily use of the device. Speech therapy is thought to be important in improving speech-recognition performance. Previous studies had demonstrated the benefits of training in speech-recognition skills of CI users and have shown promising results in adult, postlingually deafened CI recipients. However, access to rehabilitation provided by hospitals and hearing clinics is limited for many hearing-impaired patients due to time constraints, expense, and the proximity of patients to the rehabilitation site [11], which is even more serious for children under 2 years old. Many families considering CI for their children are concerned that the non-availability of speech training or other rehabilitation might make CI ineffective. To assess how important speech therapy in the rehabilitation centers is in children with early implantation, in this study, we compared CAP and SIR results between patients with speech therapy and those without.

## Methods

The protocol was approved by ethics committees of Shanghai Jiaotong University. Written informed consent was obtained from parents. The supporting Strobe Checklist is available as supporting information (Table S1).

Thirty-four congenitally profoundly deaf children who underwent CI before the age of 18 months at the Sixth Hospital affiliated with Shanghai Jiaotong University from January, 2005 to July, 2008 were included. These children were totally or almost totally deaf, and did not wear a hearing aid before or after surgery. Children with significant cognitive delay determined by Griffiths' mental development scales (<86) or with cochlear malformations presented by CT scan were excluded. 19 children received routine speech therapy in rehabilitation centers after the surgery (ST), and the remaining 15 cases did not (NST), but were exposed to the real world, as are normal hearing children. Usually, speech therapy begins with an emphasis on auditory training (detection, recognition, discrimination, and perception), followed by speech orthodontic treatment, articulation training, and language training according to the child's performance. Training took place two or three times per week and lasted 6–12 months. All children received the MED-EL COMBI 40+ cochlear implant, and the speech processing strategy during the follow-up period was continuous interleaved sampling.

For the 34 patients, the age at the time of CI ranged from 7 to 18 months (mean = 14.2 months, SD = 2.63). The ages of the children in the ST group ranged from 8 to 18 months (mean = 14.8 months, SD = 2.86), and the ages of the children in the NST group ranged from 7 to 18 months (mean = 13.95 months, SD = 2.98). The difference in age between the two groups was not significant (Student's *t*-test, *P* = 0.22).

The CAP (Table S2) was used to measure the speech perception performance of the implanted children. It measures supraliminal performance, which reflects everyday auditory performance in a more realistic way. The CAP comprises a hierarchical scale of auditory perceptive ability ranging from 0 “displays no awareness of environmental sounds” to 7 “can use the telephone with a familiar talker” [12].

The SIR (Table S3) was used to measure the speech intelligibility of the implanted children by quantifying their everyday spontaneous speech. It is a time-effective global outcome measure of speech intelligibility in real-life situations. SIR consists of five performance categories ranging from “prerecognizable words in spoken language” to “connected speech is intelligible to all listeners” [13].

All children were assessed before surgery and 6, 12, and 24 months after surgery using CAP and SIR. To eliminate test bias, the children enrolled in this study were mixed with other children who were not enrolled, and each child was rated by the same speech and language therapist who was blind to the child's situation and was not allowed to ask for information such as the duration of cochlear implant use and whether the child received speech therapy prior to scoring. The CAP and SIR scores of the groups at each time point were compared using Mann-Whitney rank-sum test, while the percentage of children at level 3 or higher at the four time points between the two groups were compared using Fisher Exact Test.

## Results

### Comparison of CAP scores of the two groups

**Figure 1:**
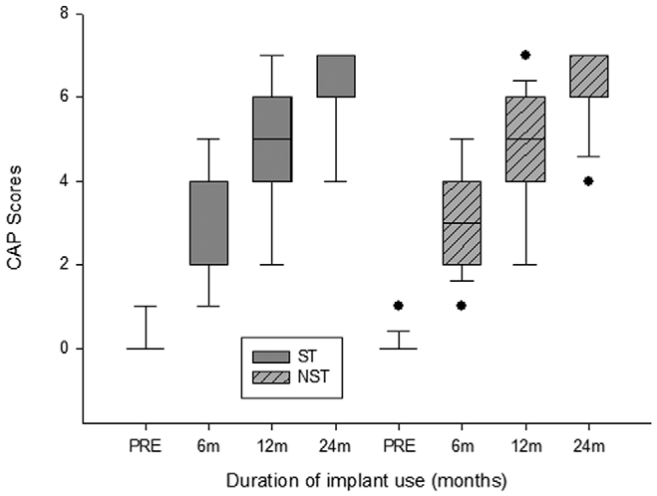
Box plot showing the distribution of CAP values of the two groups with the time of implant usage. Box plot explanation: upper horizontal line of box, 75th percentile; lower horizontal line of box, 25th percentile; horizontal bar within box, median; upper horizontal bar outside box, 90th percentile; lower horizontal bar outside box, 10th percentile. Circles represent outliers.

The median CAP scores for the two groups are shown in Figure 1. CAP scores in both groups increased with increasing time of implant use during the follow-up period after implantation, and at each time point, the median CAP score of the two groups were comparable. Before implantation, the median CAP score for the ST group was 0, and it increased to 4 at 6 months, 5 at 12 months, and 7 at 24 months after cochlear implantation. An identical change was noted for the median CAP score in the NST group. The median CAP score for that group showed that values increased from 0 at implantation to 3 at 6 months, 5 at 12 months, and 7 at 24 months. The median CAP values of the two groups were not significantly different at any point during the follow-up (Mann-Whitney rank-sum test, *P*>0.05).

The numbers and percentages of children with scores at each CAP level before, at 6 months after, and at 1 and 2 years after CI were calculated. The percentage of children at level 3 or higher increased from 0 before surgery to 73.7%, 89.5%, and 100% at 6, 12, and 24 months after implantation in the ST group, respectively; the percentages were 0 before surgery and 73.3%, 86.7%, and 100% at 6, 12, and 24 months after implantation in the NST group (Table 1). There was no significant difference in scores at the four time points between the ST and NST groups (Fisher Exact Test, P>0.05).

**Table 1:** Numbers of patients in each CAP category before implantation, 6, 12, 24 months after implantation.

CAP	Before		6 months		12 months		24 months	
Category	ST	NST	ST	NST	ST	NST	ST	NST
0	17 (89.5%)	14 (93.3%)	0 (0%)	0 (0%)	0 (0%)	0 (0%)	0 (0%)	0
1	2 (10.5%)	1 (6.7)	2 (10.5%)	1 (6.6%)	0 (0%)	0 (0%)	0 (0%)	0
2	0 (0%)	0 (0%)	3 (15.8%)	3 (20.0%)	2 (10.5%)	2 (13.3%)	0 (0%)	0
3	0 (0%)	0 (0%)	4 (21.1%)	4 (26.7%)	1 (5.3%)	1 (6.7%)	0 (0%)	0
4	0 (0%)	0 (0%)	7 (36.8%)	4 (26.7%)	3 (15.8%)	1 (6.7%)	2 (10.5%)	1 (6.7%)
5	0 (0%)	0 (0%)	3 (15.8%)	3 (20.0%)	5 (26.3%)	6 (40.0%)	2 (10.5%)	2 (13.3%)
6	0 (0%)	0 (0%)	0 (0%)	0 (0%)	6 (31.6%)	4 (26.6%)	5 (26.3%)	4 (26.7%)
7	0 (0%)	0 (0%)	0 (0%)	0 (0%)	2 (10.5%)	1 (6.7%)	10 (52.7%)	8 (53.3%)
Total	19	15	19	15	19	15	19	15

### Comparison of SIR scores between the two groups

**Figure 2:**
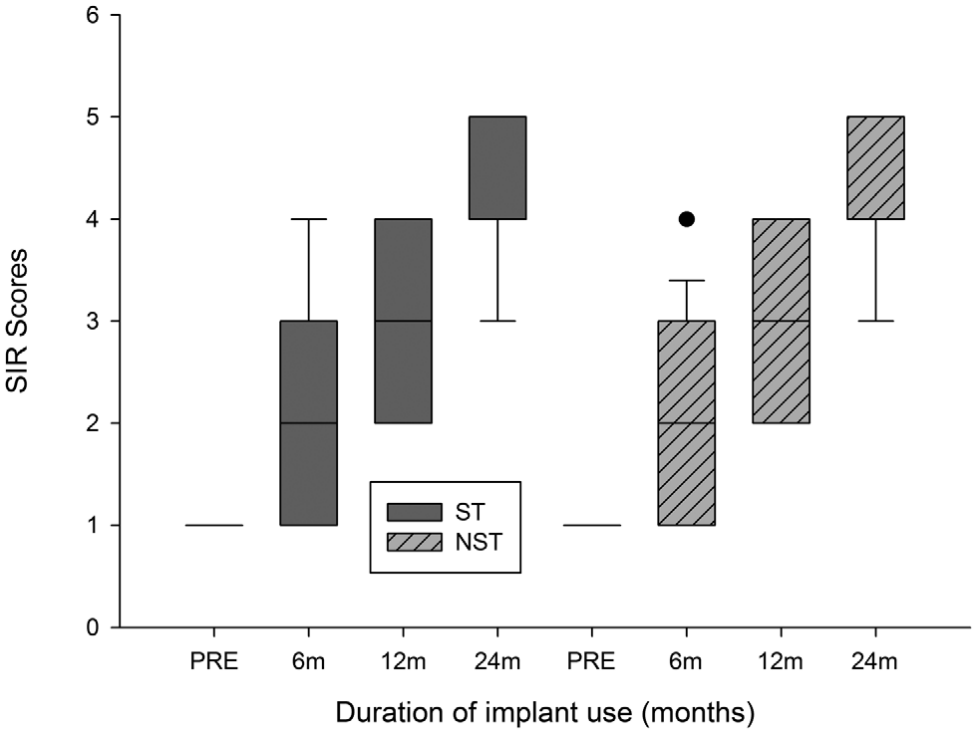
Box plot showing the distribution of SIR values of the two groups with the time of implant usage. Box plot explanation: upper horizontal line of box, 75th percentile; lower horizontal line of box, 25th percentile; horizontal bar within box, median; upper horizontal bar outside box, 90th percentile; lower horizontal bar outside box, 10th percentile. Circles represent outliers.

The median SIR values increased in a manner similar to CAP values in the two groups (Fig. 2). Before CI, the median SIR value of the ST group was 1, and it increased to 2 at 6 months, 3 at 12 months, and 5 at 24 months. For the NST group, the median SIR value was 1 before implantation and it increased to 2 at 6 months, 3 at 12 months, and 5 at 24 months. No significant difference between the groups in median SIR values was observed at any time point during the follow-up period (Mann-Whitney rank-sum test, *P*>0.05).

The numbers and percentages of children with scores at each level of SIR before, at 6 months after, and at 1 and 2 years after CI were calculated (Table 2). In this study, categories 3, 4, and 5 were all defined as intelligible, following a previous study [14]. The percentage increased from 0 before surgery to 47.4% at 6 months, 73.7% at 12 months, and 100% at 24 months after CI in the ST group, whereas in NST group, the percentages were 0 before surgery, 40% at 6 months, 73.3% at 12 months, and 100% at 24 months after CI. There was no significant difference at the four time points between the ST and NST groups (Fisher Exact Test, P>0.05).

**Table 2:** Numbers of patients in each SIR category before implantation, 6, 12, 24 months after implantation.

SIR	Before		6 months		12 months		24 months	
Category	ST	NST	ST	NST	ST	NST	ST	NST
1	19 (100%)	15 (100%)	5 (26.3%)	4 (26.7%)	0 (0%)	0 (0%)	0 (0%)	0 (0%)
2	0 (0%)	0 (0%)	5 (26.3%)	5 (33.3%)	5 (26.3%)	4 (26.7%)	0 (0%)	0 (0%)
3	0 (0%)	0 (0%)	7 (36.9%)	5 (33.3%)	6 (31.6%)	5 (33.3%)	4 (21.1%)	3 (20%)
4	0 (0%)	0 (0%)	2 (10.5%)	1 (6.7%)	8 (42.1%)	6 (40%)	2 (10.5%)	3 (20%)
5	0 (0%)	0 (0%)	0 (0%)	0 (0%)	0 (0%)	0 (0%)	13 (68.4%)	9 (60%)
Total	19	15	19	15	19	15	19	15

## Discussion

In this study, our results showed that the auditory performance and speech intelligibility of trained children in the rehabilitation centers were almost the same as those of untrained children with early implantation. After implantation, the CAP and SIR scores of both groups increased with increasing time of implant use during the follow-up period, and at each time point, the median scores of the two groups were comparable.

Speech therapy has been shown to be effective in the rehabilitation of patients with cochlear implantation in previous studies [15], [16]. It has been thought to be one of the methods that can accelerate the learning process because postlingually deafened cochlear implant patients must adapt to both spectrally reduced and spectrally shifted speech due to the limited number of electrodes and the limited length of the electrode array after implantation. Several studies have revealed better auditory resolution, speech perception, and music perception after receiving training [15], [16], suggesting great promise as part of the aural rehabilitation of adult, postlingually deafened cochlear implant recipients.

Our results may seem inconsistent with previous studies. However, our negative result was not the only one demonstrating no efficacy in CI patients receiving speech training. Mixed and generally poor outcomes have been revealed from previous CI speech-training studies [17], [18]. For example, Dawson and Clark (1997) found that three of five patients showed minimal or no improvement in vowel perception after training [18]. Most authors believe these results were due to training protocols, training materials, and training frequencies used. However, none of these factors has been confirmed, and the exact reason remains unknown.

Some studies have found that earlier implantation leads to better language outcomes, suggesting that there may be sensitive periods for central auditory and spoken language development at a younger age [19], [20]. There is considerable evidence for a developmentally sensitive period during which the auditory cortex is highly plastic, although it has not been defined clearly [10], [21]. If the auditory system is deprived of sensory input during this sensitive period, then the central auditory system is susceptible to large-scale reorganization. Very early implantation may be necessary to allow at least relatively normal organization of auditory pathways in congenitally deaf children. This might be a potential factor explaining why earlier implantation leads to good results in children even without speech training.

The length of the speech therapy for the children in this study might be insufficient to see results. A previous study reported that the generally poor outcomes of CI speech training patients might well be due to the amount and type of training used [22]. We cannot exclude this possibility. However, many studies that reported marked benefits used training protocols of less than 6 months' duration. Since they focused on adult, postlingually deafened cochlear implant recipients, comparison of these studies and ours was not proper.

The CAP and SIR are non-linear, hierarchical scales, with poor accuracy and little detail [23], and therefore they may not reveal the real conditions in these children. This may be the main limitation of the present study. However, the very young children have not established spoken language skills or the level of behavior and cooperation necessary for a more formal assessment [14]. Therefore, indirect measures may have to be used [23]. Further investigations should be undertaken using more accurate and detailed parameters and should also be conducted over a longer term with more cases. On the other hand, the benefits of ST for the early implantation children were so subtle that they could not be distinguished using real-life situation-based measures, and so might be of little clinical significance.

## Conclusions

These results indicate that great communication benefits achieved by early implantation (<18 months) without routine speech therapy, the results exemplify the importance of enhanced social environments provided by everyday life experience for human brain development and reassure parents considering cochlear implants where speech training is unavailable. Further studies should be conducted to confirm our results.

## Supporting Information

Table S1 **Strobe checklist.**
(DOC)

Table S2 **Categories of auditory performance.**
(DOC)

Table S3 **Speech intelligibility ratings.**
(DOC)
