# Supplementary material for: Categories of Auditory Performance and Speech Intelligibility Ratings of Early-Implanted Children without Speech Training
Source: PLoS One. 2013 Jan 21;8(1):e53852. doi: 10.1371/journal.pone.0053852 (PMC3549925; doi:10.1371/journal.pone.0053852)
Supplement: Table S2 — Categories of auditory performance. (DOC) [file pone.0053852.s002.doc]

**Categories of auditory performance**

0 no awareness of environmental sound

1 awareness of environmental sounds

2 responds to speech sounds

3 recognizes environmental sounds

4 discriminates at least two speech sounds

5 understands common phrases without lipreading

6 understands conversation without lipreading with a familiar talker

7 can use the telephone with a familiar talker
