# Supplementary material for: Categories of Auditory Performance and Speech Intelligibility Ratings of Early-Implanted Children without Speech Training
Source: PLoS One. 2013 Jan 21;8(1):e53852. doi: 10.1371/journal.pone.0053852 (PMC3549925; doi:10.1371/journal.pone.0053852)
Supplement: Table S3 — Speech intelligibility ratings. (DOC) [file pone.0053852.s003.doc]

**Speech intelligibility ratings**

1 Prerecognizable words in spoken language (the child’s primary mode of everyday communication may be manual)

2 Connected speech is unintelligible; intelligible speech is developing in single words when context and lip reading cues are available

3 Connected speech is intelligible to a listener who concentrates and lip-reads within a known context

4 Connected speech is intelligible to a listener who has little experience of a deaf person’s speech; the listener does not need to concentrate unduly

5 Connected speech is intelligible to all listener’s; the child is understood easily in everyday contexts
